# A Blockchain-Enabled Security Framework for Cloud-Based Sensor Systems with Deep Learning-Driven Attack Classification

**DOI:** 10.3390/s26165198

**Published:** 2026-08-17

**Authors:** Naveed Ahmad, Yue Cao, William Liu

**Affiliations:** 1 College of Computer & Information Sciences, Prince Sultan University, Riyadh 12435, Saudi Arabia; nahmed@psu.edu.sa; 2 School of Cyber Science and Engineering, Wuhan University, Wuhan 430072, China; 3 School of Computing, Electrical and Applied Technologies, Unitec Institute of Technology, Auckland 1025, New Zealand; wliu@aut.ac.nz

**Keywords:** cloud computing, cyber security, hashing, weighted, symmetric cryptography, blockchain, deep learning

## Abstract

Cloud-integrated sensor and Internet of Things (IoT) systems enable scalable data storage, processing, and intelligent monitoring, but their distributed nature exposes network-flow and host-level data to unauthorized access, tampering, and cyberattacks. This study proposes a Weighted Symmetric Hashed Blockchain (WSHB) framework that integrates mutual-information-based feature weighting, deep-learning-based attack classification, AES-256-GCM authenticated encryption, cryptographic hashing, and permissioned-ledger logging. The framework was evaluated independently using HIKARI-2021 for network-based intrusion detection and ADFA-LD for host-based intrusion detection. A transparent comparative evaluation was conducted against LSTM and CNN–RNN baselines using identical data splits, preprocessing settings, input representations, hyperparameter-search budget, and repeated initialization seeds. The proposed WSHB-DNN classifier achieved macro-F1 scores of 0.6837 on HIKARI-2021 and 0.7914 on ADFA-LD, showing the strongest overall classification performance among the evaluated models. The cryptographic and permissioned-ledger components provide confidentiality protection, record-level authentication, integrity verification, and tamper-evident logging for confirmed attack-event records. These results demonstrate the potential of WSHB as a reproducible framework for attack classification and secure event logging in cloud-integrated sensor environments.

## 1. Introduction

Cloud-integrated sensor and Internet of Things (IoT) systems have become essential components of modern digital infrastructures. These systems support large-scale data acquisition, remote monitoring, intelligent decision-making, and real-time service delivery across domains such as smart cities, healthcare, industrial automation, transportation, and cyber-physical systems. In such environments, sensor nodes continuously generate heterogeneous data streams that are transmitted through edge gateways and cloud platforms for storage, processing, analytics, and long-term management [1,2,3]. The integration of cloud computing with sensor networks provides scalable computation, flexible storage, and efficient resource management without requiring extensive local infrastructure.

Recent advances in hybrid cloud, multi-cloud, serverless computing, containerization, and edge computing have further improved the deployment and management of sensor-driven applications [4,5]. Edge computing is particularly important for sensor environments because it enables data preprocessing and near-real-time analysis close to the point of data generation. However, sensor data is often forwarded to cloud platforms for deeper analytics, machine learning, historical analysis, and centralized management. This edge–cloud integration improves system intelligence and scalability, but it also increases the security attack surface. As sensor devices are often resource-constrained and deployed in distributed environments, they are vulnerable to data tampering, unauthorized access, false data injection, denial-of-service attacks, and compromised node behavior.

Security and privacy are therefore major concerns in cloud-based sensor systems. When sensor-generated data is transmitted to external cloud platforms, organizations may lose direct control over sensitive data and operational telemetry. This creates risks related to data breaches, insider threats, insecure configurations, and unauthorized modification of sensor records [6,7,8,9]. Traditional security mechanisms such as encryption, access control, identity management, and compliance monitoring are useful, but they are not always sufficient for ensuring data integrity, provenance, and trust in distributed sensor-cloud environments. Moreover, misconfigured cloud services, exposed databases, weak access policies, and insufficient monitoring can further increase the probability of compromise.

Blockchain technology has emerged as a promising mechanism for improving trust, integrity, and accountability in distributed sensor and cloud environments. Its decentralized and tamper-resistant structure enables immutable logging of sensor data transactions, secure verification of data origin, and transparent auditing of system activities. By integrating blockchain with sensor-cloud infrastructures, it becomes possible to protect sensor data against unauthorized modification and provide stronger guarantees for data provenance and integrity. However, blockchain-based systems may introduce additional computational and latency overhead, which must be carefully considered for real-time and resource-constrained sensor applications.

Deep learning has also become an important tool for securing sensor networks and cloud-enabled IoT systems. Deep learning models can analyze complex traffic patterns, identify abnormal behavior, and classify cyberattacks with high accuracy. In sensor–cloud environments, such models can support real-time intrusion detection by learning from network traffic, system logs, and behavioral features. However, the effectiveness of deep learning-based security depends on reliable data, efficient feature extraction, and integration with secure data management mechanisms.

To address these challenges, this paper proposes a Weighted Symmetric Hashed Blockchain (WSHB) framework for cloud-integrated sensor environments. WSHB combines weighted feature analysis, symmetric encryption, cryptographic hashing, blockchain-based logging, and deep learning-based attack classification. The weighted feature analysis identifies the most security-relevant traffic attributes. Symmetric encryption protects sensitive sensor and traffic data, while hashing supports integrity verification. Blockchain is used to maintain tamper-evident records of detected attacks, and the deep learning classifier identifies malicious traffic patterns. This integrated design supports secure monitoring, attack classification, and post-incident traceability in cloud-based sensor systems. The main contributions of this study are as follows:

- A unified WSHB framework is proposed for cloud-integrated sensor and IoT systems by combining mutual-information-based feature weighting, deep learning-driven attack classification, AES-256-GCM authenticated encryption, cryptographic hashing, and permissioned-ledger logging.
- A training-derived weighted feature scoring mechanism is introduced to prioritize security-relevant attributes for anomaly screening and classification, while ensuring that the feature weights do not modify the encryption, hashing, or ledger-validation processes.
- A tamper-evident logging mechanism is developed by encrypting confirmed attack-event records and storing their cryptographic hashes in a permissioned ledger for integrity verification, authentication, and traceability.
- A transparent comparative evaluation is conducted using HIKARI-2021 for network-based intrusion detection and ADFA-LD for host-based intrusion detection against LSTM and CNN–RNN baselines. The evaluation reports complete model architectures, trainable parameter counts, hyperparameter-search settings, sequence-construction details, per-class metrics, repeated-run statistics, and statistical significance tests.

The rest of the paper is organized as follows: Section 2 discusses related work, Section 3 presents the system model, and Section 4 introduces the WSHB model. Section 5 focuses on the security analysis of the cloud environment using WSHB, while Section 6 describes the simulation environment. Finally, Section 7 provides the conclusion.

## 2. Related Work

Cloud-integrated sensor and Internet of Things (IoT) systems have become important infrastructures for smart cities, healthcare monitoring, industrial automation, transportation, and cyber-physical systems. However, the integration of sensing devices with cloud and edge platforms introduces several security challenges because sensor data is continuously transmitted, stored, and processed across distributed environments. Recent studies have examined these challenges from different perspectives, including cloud-IoT security, blockchain-assisted trust management, and machine learning or deep learning-based intrusion detection. Therefore, this section reviews the most relevant studies and identifies the research gap addressed by the proposed Weighted Symmetric Hashed Blockchain (WSHB) framework.

### 2.1. Cloud and IoT Security

Cloud-based IoT environments are exposed to several security risks, including unauthorized access, data leakage, device impersonation, denial-of-service attacks, eavesdropping, malicious traffic injection, and data tampering. Singh et al. [10] reviewed security challenges in cloud-based IoT systems and emphasized that the large number of connected devices, heterogeneous communication protocols, and dependence on external cloud services increase the attack surface of IoT deployments. Their study highlights the need for proactive security mechanisms that can protect IoT services against emerging threats in cloud-connected environments. This is directly relevant to cloud-integrated sensor systems, where sensor data must remain confidential, reliable, and traceable during transmission and storage.

Moreover, Ghaffari et al. [11] reviewed IoT security using machine learning and deep learning approaches and identified node spoofing, unauthorized data access, denial-of-service attacks, eavesdropping, and intrusion detection as major security concerns in IoT systems. Their work shows that intelligent detection mechanisms are increasingly important because conventional rule-based security methods are often insufficient for dynamic IoT traffic. However, their review also indicates that detection accuracy alone is not enough; secure data handling, integrity verification, and privacy-preserving mechanisms are also required.

Furthermore, Sajid et al. [12] proposed a hybrid machine learning and deep learning-based intrusion detection system (IDS) for detecting modern network attacks. Their work demonstrates the value of combining learning-based models for improving detection capability. However, such IDS solutions mainly focus on classification performance and do not provide immutable attack logging or cryptographic traceability. Similarly, Aljuaid and Alshamrani [13] proposed a convolutional neural network (CNN)-based IDS for cloud attack detection and reported that cloud IDS systems must handle large-scale traffic while maintaining accurate detection. These studies confirm the relevance of learning-based intrusion detection in cloud environments, but they also reveal the need for stronger integration with integrity verification, confidentiality protection, and audit mechanisms.

In addition, recent work has further emphasized the need for advanced IDS mechanisms in cloud–edge IoT infrastructures. Assiri et al. [14] reviewed advanced IDS approaches for cloud–edge IoT systems and highlighted that emerging IoT deployments require scalable, adaptive, and intelligent security models. Their work supports the argument that cloud-integrated sensor environments require security solutions that go beyond isolated detection models and consider data protection, traceability, and operational scalability together.

### 2.2. Blockchain-Based Security for Cloud and IoT

Blockchain has been widely investigated as a security mechanism for IoT and cloud environments because of its decentralization, immutability, transparency, and tamper resistance. Almarri and Aljughaiman [15] conducted a systematic literature review on blockchain technology for IoT security and trust. Their review explains that blockchain can support secure identity management, data integrity, trust establishment, and tamper-resistant transaction recording in IoT systems. These properties are important for cloud-based sensor environments because sensor records may be modified, deleted, or manipulated if stored only in centralized systems.

Moreover, Shalabi et al. [16] presented a systematic review of blockchain-based intrusion detection and prevention systems in IoT networks. Their study shows that blockchain can improve IDS and intrusion prevention systems by supporting decentralized attack record management, secure sharing of detection results, and protection against manipulation of security logs. However, the review also notes that blockchain-based IDS solutions may introduce latency, computational overhead, and scalability challenges. Therefore, blockchain must be integrated carefully with lightweight cryptographic and learning-based mechanisms when applied to sensor and IoT systems.

Furthermore, Ali et al. [17] reviewed blockchain- and federated learning-based intrusion detection systems for Industrial IoT networks. Their study highlights that blockchain can strengthen privacy, trust, and reliability in collaborative IDS environments, while federated learning can reduce the need to share raw data. Although this direction is promising, many existing frameworks focus on distributed learning and collaborative detection rather than combining cryptographic hashing, symmetric encryption, blockchain logging, and weighted traffic classification in a single workflow.

Recent studies have also explored the combined use of blockchain and machine learning for IoT intrusion detection. Sathyabama and Katiravan [18] proposed an integrated anomaly detection and prevention framework using deep neural networks and blockchain for IoT cybersecurity. Their framework uses deep neural networks for anomaly detection and blockchain for data integrity, transparency, decentralized security, and tamper-resistant logging. This study is closely related to the proposed WSHB framework because it also combines intelligent attack detection with blockchain-based security. However, WSHB further integrates weighted feature analysis, symmetric encryption, and hash-based verification before blockchain logging.

Similarly, Song et al. [19] proposed a hybrid blockchain and machine learning-based intrusion detection system for Industrial IoT environments. Their framework uses blockchain to ensure data integrity, secure communication, and resistance against unauthorized modification, while machine learning is used to improve threat detection accuracy. Although their work validates the usefulness of blockchain-assisted IDS in Industrial IoT, it does not explicitly combine weighted feature scoring, symmetric encryption, cryptographic hashing, and blockchain-based traceability within one cloud-integrated sensor workflow.

In the same direction, Kumar et al. [20] presented a secure blockchain-based intrusion detection model for IoT networks. Their model integrates blockchain with IDS to support data integrity, decentralization, tamper-proof intrusion logging, trust, transparency, and real-time threat detection. Compared with this work, the proposed WSHB framework focuses on a unified security pipeline that combines feature weighting, symmetric encryption, hashing, blockchain logging, and deep learning-based attack classification for cloud-integrated sensor systems.

### 2.3. Deep Learning-Based Intrusion Detection

Deep learning has become an effective approach for intrusion detection because it can learn complex traffic patterns and identify nonlinear relationships between network features. Khan et al. [21] proposed a hybrid deep learning IDS for IoT networks using recurrent neural networks and gated recurrent units. Their model was evaluated on the ToN-IoT dataset and showed the potential of recurrent architectures for detecting attacks across IoT layers. However, such models mainly focus on detection accuracy and do not provide cryptographic integrity verification or tamper-evident attack logging.

Furthermore, Aljuaid and Alshamrani [13] proposed a CNN-based intrusion detection model for cloud environments, showing that convolutional architectures can improve cyberattack detection in large-scale traffic settings. Similarly, Selem et al. [22] proposed an ensemble deep learning IDS that combines deep neural networks and convolutional neural networks to improve detection accuracy and resilience against diverse IoT threats. These studies demonstrate that deep learning architectures can improve attack classification performance, especially when traffic behavior is complex and heterogeneous.

Moreover, Afraji et al. [23] introduced an integrated hybrid deep learning framework for intrusion detection in IoT and Industrial IoT networks using CNN, long short-term memory (LSTM), and gated recurrent unit (GRU) architectures. Their model captures spatial and temporal dependencies in network traffic and evaluates CNN-LSTM, CNN-GRU, and CNN-LSTM-GRU models for binary and multiclass intrusion detection. However, their approach mainly focuses on classification performance and does not provide cryptographic integrity verification or tamper-evident blockchain logging.

In addition, Eshmawi et al. [24] proposed a ConvLSTM-based threat detection framework for Industrial IoT and cloud computing environments. Their work combines the spatial feature extraction capability of CNNs with the temporal learning capability of LSTM models to predict and mitigate threats in IoT, Industrial IoT, and cloud environments. This supports the view that deep learning models are suitable for complex traffic behavior in cloud-connected IoT systems. Nevertheless, the framework does not provide the same level of integration between encryption, hashing, weighted feature scoring, and blockchain-based attack traceability as the proposed WSHB framework.

More recently, Mohan and Shanmugavel [25] proposed ADeepCRF, a real-time threat detection framework for IoT networks using blockchain-based advanced proof of authority and adaptive deep conditional random fields. Their work integrates blockchain for tamper-proof and decentralized threat information management and uses adaptive deep conditional random fields for threat detection. This recent study confirms the growing importance of integrating blockchain with advanced learning models for IoT security. Compared with this approach, WSHB emphasizes a structured security workflow based on weighted feature analysis, symmetric encryption, hash verification, blockchain logging, and deep learning-based classification.

The reviewed studies show that recent cloud-IoT security, blockchain-assisted IDS, and deep learning-based intrusion detection approaches provide useful foundations for WSHB. Cloud-IoT security studies identify the main threats and deployment challenges. Blockchain-based studies support the use of tamper-evident logging, integrity verification, and decentralized trust. Deep learning-based IDS studies justify the use of learning models for attack classification. However, these studies usually address detection, encryption, hashing, and blockchain logging as separate components. The proposed WSHB framework connects these components in a single workflow by linking weighted traffic analysis, AES-based encryption, hash-based integrity verification, blockchain logging, and deep learning-based attack classification.

## 3. System Model

In this model, the cloud computing environment incorporates the idea of blockchain for security, data integrity, and trust management throughout the services. The components of the system include cloud infrastructure, blockchain ledger, smart contract, cryptographic algorithms, virtual machines (VMs), storage, networking, and various cloud services such as IaaS, PaaS, and SaaS. Let the set of cloud resources be represented as shown in Equation (1):

(1) $$\mathrm{CCI}=\left\{{\mathrm{VM}}_{1},{\mathrm{VM}}_{2},\ldots ,{\mathrm{VM}}_{n}\right\}.$$

Each ${\mathrm{VM}}_{i}$ has its roles to perform, for performing calculations, deciding file locations, or deploying applications. The cloud structure is multitenant, hence it supports the sharing of physical infrastructure, though the tenants’ workload is kept separate. Blockchain has the role of the distributed and tamper-proof ledger for maintaining records of transactions, smart contracts, and audits. It uses a tree and each tree node holds one copy of the Blockchain ledger. The key elements in this model include the Blockchain Ledger *L*, a chain of blocks linked with each other and containing transactional data along with proofs in the form of cryptocurrencies, defined in Equation (2):

(2) $$L=\{B_{1},B_{2},\ldots ,B_{m}\}$$

where each block $B_{i}$ contains the following:

(3) $$B_{i}=\left\{H\left(M_{i}\right),T_{i},H_{\mathrm{prev}}\left(B_{i-1}\right)\right\}$$

In Equation (3), $H(M_{i})$ is the hash of the message or the data, $T_{i}$ is the timestamp, and $H_{\mathrm{prev}}\left(B_{i-1}\right)$ is the hash of the previous block to make it more immutable.

Transactions are validated with a reliable consensus mechanism such as Proof of Work and Proof of Stake among many others used for consistency of nodes. Let *C* represent the level of the consensus function stated in Equation (4):

(4) $$C=f(B_{1},B_{2},\ldots ,B_{m})$$

The function confirms that the process and each transaction recorded within the blockchain are authentic and that all members are synchronized with the ledger.

The smart contract is a self-executing digital contract that deals with conditions to implement those terms when copied out by a set of codes. The cloud environment is a place where smart contracts can be employed, and these tasks can include or involve resource orchestration, cost calculation, or permission rights of the data, respectively. To this, let SC represent the set of smart contracts in the system stated in Equation (5):

(5) $$SC=\{SC_{1},SC_{2},\ldots ,SC_{k}\}$$

The smart contract $SC_{i}$ will only be activated upon triggering some conditions. For instance, the criteria of the resource provisioning smart contract can be described in Equation (6):

(6) $$SC_{i}\left(\mathrm{Resource},\mathrm{Condition}\right)\;\mathrm{if}\;\mathrm{Condition}\;\mathrm{is}\;\mathrm{True}$$

The smart contract performs specific actions like providing access to an interface of a VM or allocating more storage.

Concerning the presence of data in the cloud, data authenticity should be preserved at the utmost level. Based on the analysis, it was understandable that blockchain is to establish a decentralized platform to validate the data integrity across the distributed cloud resources. Every transaction and data change is recorded onto the blockchain to make for a more transparent ledger. The user shares data on the cloud, the transaction is recorded in the blockchain as a set of blocks and the data cannot be changed thereafter.

**Figure 1:**
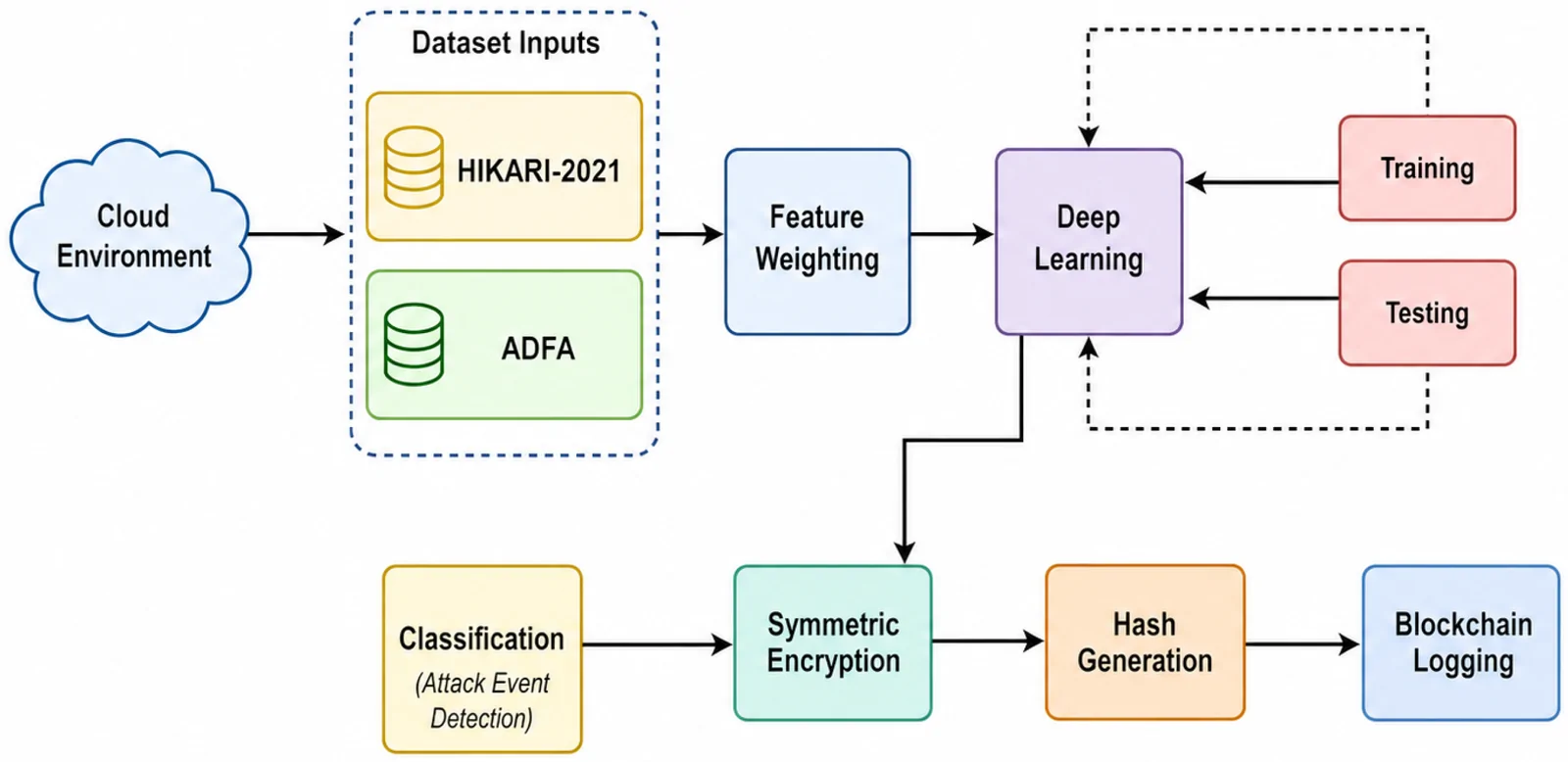
Process in proposed WSHB.

Figure 1 presents the flow of the proposed WSHB model for cybersecurity attack detection and classification.

### 3.1. Datasets

The WSHB framework was evaluated independently using HIKARI-2021 for network-based intrusion detection and ADFA-LD for host-based intrusion detection. Because the two datasets contain different data representations, dataset-specific preprocessing was applied before classification through the common WSHB pipeline.

#### 3.1.1. HIKARI-2021

HIKARI-2021 contains labelled network-flow records described by source and destination addresses, transport ports, protocols, flow duration, and packet- and byte-level statistics. The ALLFLOWMETER_HIKARI2021.csv file was used. Duplicate records were removed before sampling, and a stratified subset of 10,000 records was generated using the fixed random seed 42. Missing numerical values were imputed using the training-subset median, categorical attributes were encoded, and numerical attributes were scaled using min–max normalization. All preprocessing parameters were estimated exclusively from the training subset and subsequently applied to the validation and testing subsets. For binary classification, label 0 was mapped to the benign class and label 1 was mapped to the attack class.

#### 3.1.2. ADFA-LD

ADFA-LD contains ordered Linux system-call traces generated during normal host operations and malicious activities. Unlike HIKARI-2021, it does not contain network-flow attributes such as IP addresses, transport ports, packet counts, or flow duration. A stratified subset of 5000 traces was selected from the official ADFA-LD archive using the fixed random seed 42. The system-call identifiers in each trace were converted into fixed-length numerical vectors before feature weighting and classification. For binary classification, normal traces were mapped to the benign class, whereas traces contained in the attack directories were mapped to the attack class.

The two datasets were divided independently into 70% training, 15% validation, and 15% testing subsets. Sampling was performed before the final data split, and the same split proportions were maintained for both datasets as shown in Table 1.

## 4. Weighted Symmetric Hashed Blockchain (WSHB)

The proposed Weighted Symmetric Hashed Blockchain (WSHB) framework integrates feature weighting, anomaly screening, deep learning-based attack classification, symmetric encryption, cryptographic hashing, and permissioned-ledger logging to support secure monitoring in cloud-based sensor environments. In the revised framework, each component has a distinct role. Feature weighting and anomaly screening are used to identify suspicious records, the deep neural network is used to determine the final attack label, and the cryptographic and ledger components are used to protect and record confirmed attack events in a tamper-evident manner.

To avoid ambiguity, the WSHB framework follows one consistent processing order: data preprocessing, mutual-information-based feature weighting, anomaly screening, deep learning-based final attack classification, symmetric encryption of the confirmed attack-event record, cryptographic hash generation, and permissioned-ledger logging. The feature weights are used only for anomaly screening and classification; they do not modify the symmetric encryption or hashing algorithms.

The following subsections describe the end-to-end WSHB pipeline, the feature-weighting and anomaly-screening stage, the deep learning classifier, and the cryptographic and ledger-logging process in detail.

### 4.1. End-to-End WSHB Processing Pipeline

The proposed WSHB framework follows a single and sequential processing pipeline consisting of data preprocessing, mutual-information-based feature weighting, anomaly screening, deep learning-based attack classification, cryptographic protection, and permissioned-ledger logging. The complete processing order is illustrated in Figure 1.

During the training phase, the dataset is first divided into training, validation, and testing subsets. All preprocessing parameters, including missing-value imputation, categorical encoding, and numerical normalization, are learned exclusively from the training subset. Mutual-information scores are also calculated using only the training subset and are normalized to obtain fixed feature weights. These learned preprocessing parameters and feature weights are subsequently applied to the validation and testing subsets without re-estimation.

During the inference phase, an incoming traffic record is preprocessed using the parameters learned from the training data. The fixed feature weights are then applied to construct a weighted feature vector and calculate an anomaly screening score. The anomaly score is used only as a preliminary screening mechanism. If the score exceeds the predefined threshold, the weighted feature vector is forwarded to the trained deep neural network, which produces the final attack label.

After the DNN confirms an attack, the framework constructs an attack-event record containing the record identifier, timestamp, predicted attack label, anomaly score, and selected event metadata. The attack-event record is first encrypted using the symmetric encryption mechanism. A cryptographic hash is then calculated over the encrypted record and its associated metadata. Finally, the encrypted record, generated hash, predicted attack label, anomaly score, timestamp, and previous-block hash are appended to the permissioned simulation ledger.

Feature weights are used exclusively in the anomaly-screening and deep learning classification stages. They do not modify the symmetric encryption algorithm, encryption key, cryptographic hash function, or ledger-verification procedure. Only DNN-confirmed attack events are cryptographically protected and recorded in the ledger, whereas records that do not exceed the anomaly threshold continue through the normal monitoring process.

### 4.2. Mutual-Information Feature Weighting and Anomaly Screening

Let

(7) $$X_{i}=\left(x_{i1},x_{i2},\ldots ,x_{in}\right)$$

represent the preprocessed feature vector of the *i*-th traffic record, where $x_{ij}$ is the normalized value of the *j*-th feature and *n* is the total number of input features.

In the proposed framework, mutual information is used as the sole feature-weighting method. The mutual-information score between feature $x_{j}$ and the class label *y* is denoted by $I(x_{j};y)$. The normalized weight of feature $x_{j}$ is calculated as

(8) $$w_{j}=\frac{I(x_{j};y)}{\sum_{k=1}^{n}I\left(x_{k};y\right)}.$$

The mutual-information scores and the corresponding normalized weights are calculated exclusively from the training subset to prevent information leakage. Once learned, the weights remain fixed and are applied without re-estimation to the validation, testing, and incoming traffic records.

The weighted representation of the *i*-th traffic record is defined as

(9) $$X_{i}^{w}=\left(w_{1}x_{i1},w_{2}x_{i2},\ldots ,w_{n}x_{in}\right).$$

The anomaly-screening score for the *i*-th record is calculated as

(10) $$S_{i}=\sum_{j=1}^{n}w_{j}x_{ij}.$$

The mean and standard deviation of the anomaly scores are estimated using the training subset only:

(11) $$\mu_{S}=\frac{1}{N_{\mathrm{tr}}}\sum_{i=1}^{N_{\mathrm{tr}}}S_{i},$$

(12) $$\sigma_{S}=\sqrt{\frac{1}{N_{\mathrm{tr}}}\sum_{i=1}^{N_{\mathrm{tr}}}{\left(S_{i}-\mu_{S}\right)}^{2}}.$$

The anomaly-screening threshold is defined as

(13) $$T=\mu_{S}+k\sigma_{S},$$

where *k* is a sensitivity parameter selected using the validation subset.

The screening and classification process is expressed as

(14) $$S_{i}>T\;⟹\;{\hat{y}}_{i}=f_{\theta }\left(X_{i}^{w}\right),$$

where $f_{\theta }\left(\cdot \right)$ denotes the trained deep neural network and ${\hat{y}}_{i}$ is the final predicted attack label.

The anomaly score is not used as the final attack label. It determines only whether a record is forwarded to the trained DNN for final classification. Furthermore, the feature weights do not influence the cryptographic stage. They are not used to generate the encryption key, modify the symmetric encryption operation, select the hashing algorithm, or determine the ledger-validation process.

### 4.3. Deep Learning-Based Attack Classification

After weighted feature scoring, the detected suspicious records are processed by the deep learning-based attack classification module. The objective of this module is to classify each traffic record as benign or malicious and, in the case of malicious traffic, identify the corresponding attack category. Let $X_{i}^{w}=w_{1}x_{i1},w_{2}x_{i2},\ldots ,w_{n}x_{in}$ represent the weighted feature vector of the *i*-th traffic record, where $x_{ij}$ is the value of the *j*-th feature and $w_{j}$ is its corresponding normalized weight.

The classifier is implemented as a feed-forward deep neural network for structured network-traffic features. The input layer receives the normalized weighted feature vector. Three dense hidden layers with 128, 64, and 32 neurons are used to learn nonlinear relationships among traffic attributes. The rectified linear unit (ReLU) activation function is applied in the hidden layers, and dropout regularization is used to reduce overfitting. For binary classification, the output layer uses a sigmoid activation function, whereas for multiclass attack classification, the output layer uses a softmax activation function.

The classifier learns a mapping function $f_{\theta }\left(.\right)$ from the weighted feature vector to the predicted class label:

(15) $${\hat{y}}_{i}=f_{\theta }\left(X_{i}^{w}\right),$$

where θ represents the trainable parameters of the model and ${\hat{y}}_{i}$ is the predicted class label for the *i*-th traffic record. For multiclass classification, the probability of class *c* is computed as

(16) $$P\left(y_{i}=c|X_{i}^{w}\right)=\frac{\exp(z_{c})}{\sum_{r=1}^{C}\exp\left(z_{r}\right)},$$

where *C* is the total number of classes and $z_{c}$ is the logit value associated with class *c*. The model is trained by minimizing the categorical cross-entropy loss:

(17) $$L=-\frac{1}{N}\sum_{i=1}^{N}\sum_{c=1}^{C}y_{ic}\log\left({\hat{y}}_{ic}\right),$$

where *N* is the number of training samples, $y_{ic}$ is the true class indicator, and ${\hat{y}}_{ic}$ is the predicted probability for class *c*.

For the reproducible comparative evaluation, the WSHB-DNN classifier was optimized using Adam with class-weighted binary cross-entropy. A batch size of 256 was used for all evaluated models to ensure a consistent training protocol. Hyperparameter tuning was performed on the validation subset using an equal search budget for WSHB-DNN, LSTM, and CNN–RNN. The candidate configurations varied the learning rate, dropout rate, and model-specific parameters, and the final configuration was selected based on the highest validation macro-F1 score.

During tuning, each model was trained for up to 15 epochs with early stopping patience of 3. For the final repeated-run evaluation, the selected configuration was trained for up to 30 epochs with early stopping patience of 5. The datasets were divided into training, validation, and testing subsets using a 70:15:15 split. The training subset was used to learn model parameters and preprocessing statistics, the validation subset was used for hyperparameter selection, threshold calibration, and early stopping, and the testing subset was reserved exclusively for final evaluation.

During training, the DNN is optimized using the labelled weighted feature vectors from the training subset. The validation subset is used for early stopping, hyperparameter selection, and model selection, whereas the testing subset is reserved exclusively for final performance evaluation.

During inference, only records whose anomaly scores exceed the screening threshold are forwarded to the trained DNN. The final attack label is produced exclusively by the DNN output. No additional signature-matching or rule-based classifier is used to determine the final attack category.

### 4.4. Authenticated Encryption and Permissioned-Ledger Logging

After the DNN confirms that a suspicious record belongs to an attack class, the framework constructs an attack-event record $M_{i}$ as

(18) $$M_{i}=\left\{r_{i},t_{i},{\hat{y}}_{i},S_{i},m_{i}\right\},$$

where $r_{i}$ is the unique record identifier, $t_{i}$ is the event timestamp, ${\hat{y}}_{i}$ is the DNN-predicted attack label, $S_{i}$ is the anomaly score, and $m_{i}$ contains the selected event metadata.

The proposed framework uses AES-256 in Galois/Counter Mode (AES-256-GCM) for authenticated encryption. A versioned 256-bit key $K^{\left(v\right)}$ is used, where *v* denotes the key-version identifier. For every confirmed attack-event record, a fresh and unique 96-bit nonce $N_{i}$ is generated. The additional authenticated data is defined as

(19) $$A_{i}=\mathrm{Encode}\left(r_{i},t_{i},{\hat{y}}_{i},S_{i},h_{i-1},v\right),$$

where $h_{i-1}$ is the hash of the preceding block and Encode(·) represents deterministic serialization.

AES-256-GCM produces the ciphertext $C_{i}$ and a 128-bit authentication tag $\tau_{i}$ as

(20) $$\left(C_{i},\tau_{i}\right)={\mathrm{Enc}}_{K^{\left(v\right)}}^{\mathrm{GCM}}\left(N_{i},M_{i},A_{i}\right).$$

During decryption, the authentication tag is verified before the plaintext is released:

(21) $$M_{i}={\mathrm{Dec}}_{K^{\left(v\right)}}^{\mathrm{GCM}}\left(N_{i},C_{i},A_{i},\tau_{i}\right).$$

If authentication-tag verification fails, the record is rejected. The nonce is stored with the ciphertext and is never reused under the same key version.

The encryption keys are generated and maintained by an access-controlled key-management component and are released only to authenticated WSHB modules. A fresh key version is generated for each independent experimental run. The ledger stores only the key-version identifier and never stores the encryption key itself. If a key is suspected to be compromised, that version is revoked and disabled for subsequent encryption operations, and a new key version is activated.

Hash generation is performed after authenticated encryption. The hash of the *i*-th ledger entry is calculated as

(22) $$\begin{array}{cc} h_{i}=H( & i\|t_{i}\|r_{i}\|N_{i}\left\|C_{i}\right\|\tau_{i}\\ & \|{\hat{y}}_{i}\|S_{i}\left\|v\right\|h_{i-1}), \end{array}$$

where H(·) is the cryptographic hash function and ‖ denotes deterministic ordered concatenation.

The corresponding ledger block is represented as

(23) $$B_{i}=\left\{i,t_{i},r_{i},N_{i},C_{i},\tau_{i},{\hat{y}}_{i},S_{i},v,h_{i-1},h_{i}\right\}.$$

Before appending the block, the framework verifies the AES-GCM authentication tag, recomputes the block hash, and checks the linkage with the preceding block. The block is considered valid only when

(24) $$\mathrm{VerifyTag}\left(K^{\left(v\right)},N_{i},C_{i},A_{i},\tau_{i}\right)=1,$$

(25) $$h_{i}^{\prime }=h_{i}\;\wedge \;B_{i}.h_{i-1}=B_{i-1}.h_{i}.$$

A failure in authentication-tag verification, block-hash verification, or previous-block linkage causes the ledger entry to be rejected. Thus, AES-256-GCM provides confidentiality and record-level authentication, while the block hash and previous-hash linkage provide ledger-level tamper evidence.

### 4.5. WSHB Processing Algorithm

Algorithm 1 summarizes the complete training, inference, classification, cryptographic-protection, and ledger-logging procedure.
**Algorithm 1** End-to-end WSHB processing pipeline**Require:** Training subset $D_{\mathrm{tr}}$, validation subset $D_{\mathrm{val}}$, incoming record $x_{i}$, and active key version $K^{\left(v\right)}$**Ensure:** Screening result, predicted label ${\hat{y}}_{i}$, and verified ledger entry**Training phase**  1:Fit missing-value handling, categorical encoding, and normalization using $D_{\mathrm{tr}}$ only  2:Transform $D_{\mathrm{tr}}$ and $D_{\mathrm{val}}$ using the training-derived parameters  3:Calculate mutual-information scores $I(x_{j};y)$ using $D_{\mathrm{tr}}$  4:Normalize the mutual-information scores to obtain fixed weights $w_{j}$  5:Construct weighted training vectors $X_{i}^{w}$  6:Calculate the training anomaly scores and estimate threshold *T*  7:Train the DNN $f_{\theta }\left(\cdot \right)$ using labelled weighted records  8:Use $D_{\mathrm{val}}$ for early stopping and model selection**Inference and secure logging phase**  9:Preprocess incoming record $x_{i}$ using the training-derived parameters10:Apply the fixed feature weights to obtain $X_{i}^{w}$11:Calculate the anomaly score $S_{i}$12:**if** $S_{i}\leq T$ **then**13:    **return** benign screening outcome and continue monitoring14:**else**15:    Predict the final attack label ${\hat{y}}_{i}\leftarrow f_{\theta }\left(X_{i}^{w}\right)$16:    Construct the attack-event record $M_{i}\leftarrow \left\{r_{i},t_{i},{\hat{y}}_{i},S_{i},m_{i}\right\}$17:    Retrieve the previous-block hash $h_{i-1}$18:    Retrieve the active 256-bit key $K^{\left(v\right)}$ from the access-controlled key-management component19:    **if** $\mathrm{Status}(K^{\left(v\right)})\neq \mathrm{active}$ **then**20:        Reject the cryptographic operation21:        **return** key-management error22:    **end if**23:    Generate a fresh 96-bit nonce $N_{i}\leftarrow \mathrm{CSPRNG}\left(96\right)$24:    Construct the additional authenticated data$$A_{i}\leftarrow \mathrm{Encode}\left(r_{i},t_{i},{\hat{y}}_{i},S_{i},h_{i-1},v\right)$$25:    Perform AES-256-GCM authenticated encryption$$\left(C_{i},\tau_{i}\right)\leftarrow {\mathrm{Enc}}_{K^{\left(v\right)}}^{\mathrm{GCM}}\left(N_{i},M_{i},A_{i}\right)$$26:    Calculate the current-block hash$$h_{i}\leftarrow H(i\|t_{i}\|r_{i}\|N_{i}\|C_{i}\|\tau_{i}\|{\hat{y}}_{i}\|S_{i}\left\|v\right\|h_{i-1})$$27:    Construct the ledger block$$B_{i}\leftarrow \left\{i,t_{i},r_{i},N_{i},C_{i},\tau_{i},{\hat{y}}_{i},S_{i},v,h_{i-1},h_{i}\right\}$$28:    Verify the AES-GCM authentication tag29:    Recompute $h_{i}^{\prime }$ and verify the previous-block linkage30:    **if** tag verification succeeds, $h_{i}^{\prime }=h_{i}$, and $B_{i}\cdot h_{i-1}=B_{i-1}\cdot h_{i}$ **then**31:        Append $B_{i}$ to the permissioned ledger32:    **else**33:        Reject the ledger entry and report an integrity error34:    **end if**35:**end if**36:**return** screening outcome, ${\hat{y}}_{i}$, and ledger-verification result

## 5. Security Analysis in Cloud Environment with WSHB

The security analysis of the proposed WSHB framework focuses on confidentiality, integrity verification, tamper-evident logging, and attack classification under the selected dataset-based simulation setting. AES-256-GCM authenticated encryption is used to protect confirmed attack-event records and verify their authenticity before decryption. Blockchain logging is used to preserve detected attack events in a tamper-evident form. The deep learning classifier supports attack classification using weighted traffic features. However, the current evaluation is limited to dataset-based network-flow and host-level intrusion-detection scenarios using HIKARI-2021 and ADFA-LD.

### Threat Model and Security Assumptions

The adversary is assumed to generate malicious network-flow activity in the HIKARI-2021 setting and malicious host-level system-call behavior in the ADFA-LD setting. The adversary may also attempt to modify stored attack-event records after detection. WSHB aims to detect malicious traffic, encrypt sensitive records, verify data integrity, and store attack logs in a tamper-evident blockchain ledger. The current evaluation does not consider compromised encryption keys, compromised blockchain majority, consensus-level attacks, fully compromised cloud providers, or privacy leakage from blockchain metadata. Large-scale latency under real-world deployment is also outside the scope of this study. Therefore, claims related to immutability, tamper resistance, scalability, and attack mitigation are limited to the selected simulation setting.

**Figure 2:**
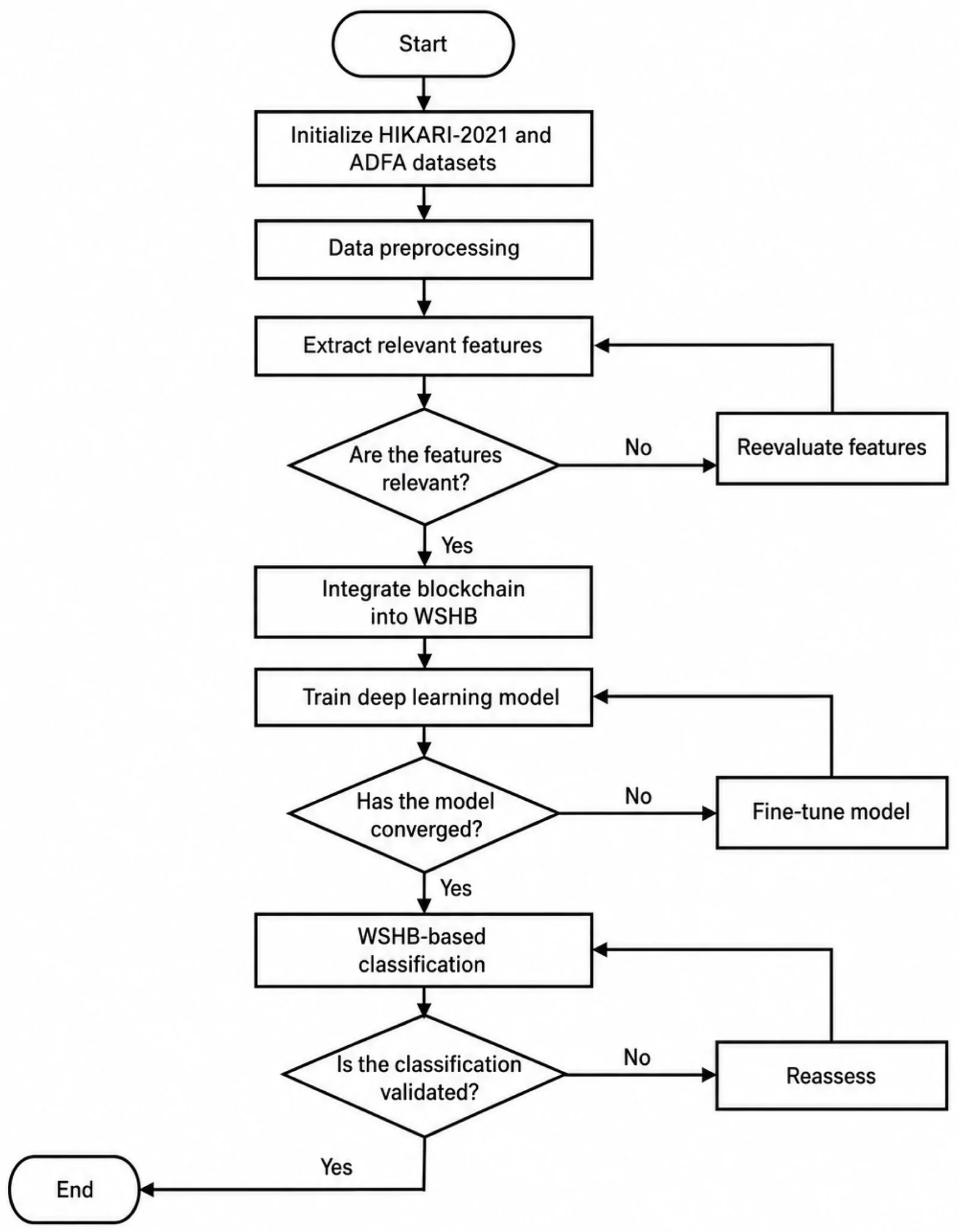
Flow chart of WSHB for the cyberattack classification.

The complete WSHB processing pipeline, including mutual-information-based feature weighting, anomaly screening, DNN-based attack classification, cryptographic protection, and permissioned-ledger logging, is described in Section 4. This section does not redefine the processing workflow; instead, it analyzes the security properties, adversarial assumptions, and limitations of the proposed framework, as shown in Figure 2.

Under the stated simulation assumptions, WSHB uses symmetric encryption to protect confirmed attack-event records, cryptographic hashing to identify unauthorized modifications, and previous-hash linkage to provide tamper-evident ledger verification. The security analysis is limited to the implemented permissioned simulation environment and does not assume resistance against compromised cryptographic keys, malicious validator majorities, consensus-level attacks, or fully compromised cloud providers.

Accordingly, the security claims in this study are limited to the implemented permissioned simulation setting. WSHB provides tamper-evident logging by combining AES-GCM authentication, SHA-256 hashing, and previous-hash linkage; however, it does not prove immutability against malicious validators, validator collusion, consensus-level attacks, replay attacks, key compromise, or metadata leakage. Similarly, scalability and deployment-latency claims are limited to the reported simulation results and should not be interpreted as evidence of large-scale production deployment.

## 6. Simulation Environment

The experimental environment evaluates the WSHB framework under two independent intrusion-detection settings. HIKARI-2021 provides labelled network-flow records for network-based intrusion detection, whereas ADFA-LD provides Linux system-call traces for host-based intrusion detection. The two datasets are therefore processed separately using dataset-specific preprocessing and feature-representation procedures.

The experiments were conducted in a dataset-driven Python 3.14 simulation environment representing cloud, IoT, host, IDS, and permissioned-ledger components. For the HIKARI-2021 experiment, encoded and normalized network-flow records were used to simulate network-monitoring events. For the ADFA-LD experiment, numerical representations of system-call traces were used to simulate host-level monitoring events. ADFA-LD was not treated as a network-flow dataset, and network attributes such as IP addresses, transport ports, packet counts, and flow duration were not used in its evaluation.

In both settings, each preprocessed record was processed through the mutual-information-based feature-weighting and anomaly-screening stages. Records whose anomaly scores exceeded the selected threshold were forwarded to the trained DNN for final classification. Records classified as benign continued through the normal monitoring process. For each DNN-confirmed attack, an attack-event record was constructed and protected using AES-256-GCM authenticated encryption. A cryptographic hash was then calculated over the ciphertext and associated metadata, and the resulting entry was appended to the permissioned simulation ledger.

The simulation used multiple virtual nodes to represent cloud servers, IoT or network nodes, monitored hosts, IDS components, and ledger nodes. The virtual-node configuration was used to evaluate classification performance, cryptographic-processing overhead, block-creation time, verification time, integrity checking, and transaction throughput under the selected dataset-based settings. The complete dataset-specific simulation and cryptographic configuration is reported in Table 2.

### 6.1. Implementation and Reproducibility Details

The WSHB framework was implemented in a Python-based simulation environment using TensorFlow/Keras for deep learning models and Scikit-learn for preprocessing, feature weighting, and performance evaluation. The experimental workflow comprised dataset acquisition, dataset-specific preprocessing, mutual-information-based feature weighting, DNN-based attack classification, authenticated encryption, permissioned-ledger logging, and performance evaluation. HIKARI-2021 and ADFA-LD were evaluated independently using a 70:15:15 split for training, validation, and testing, respectively. All preprocessing parameters, feature weights, and model hyperparameters were estimated using the training or validation subsets only, while the testing subset was reserved for final evaluation.

For HIKARI-2021, duplicate records were removed, missing numerical and categorical values were handled using training-subset median and mode imputation, respectively, categorical attributes were encoded, and numerical network-flow features were scaled using min–max normalization. ADFA-LD system-call traces were processed separately and converted into numerical sequence representations before feature weighting and classification. Network-flow attributes, including IP addresses, ports, packet counts, and flow duration, were not used in the ADFA-LD experiment. Mutual-information scores were computed exclusively from each training subset and normalized to produce fixed feature weights. Where class imbalance was observed, class-weighted loss was used to reduce bias toward majority classes.

The classifier was implemented as a feed-forward deep neural network with three dense hidden layers containing 128, 64, and 32 neurons. ReLU activation and dropout regularization were applied in the hidden layers. For the reproducible comparative evaluation, WSHB-DNN, LSTM, and CNN–RNN were trained using Adam optimization, class-weighted binary cross-entropy, a batch size of 256, and early stopping. Hyperparameter tuning was performed on the validation subset using an equal search budget for all evaluated models. The candidate configurations varied the learning rate, dropout rate, and model-specific parameters, and the final configuration was selected using validation macro-F1. During tuning, each model was trained for up to 15 epochs with early-stopping patience of 3. For the final repeated-run evaluation, the selected configuration was trained for up to 30 epochs with early-stopping patience of 5. The testing subset was reserved exclusively for final evaluation.

For cryptographic processing, confirmed attack-event records were protected using AES-256-GCM with a 256-bit versioned key, a fresh 96-bit nonce for each encryption operation, and a 128-bit authentication tag. Encryption time included nonce generation, ciphertext generation, and tag generation, whereas decryption time included plaintext recovery and authentication-tag verification. Keys were maintained by an access-controlled key-management component, and a fresh key version was activated for each independent experimental run.

The blockchain component was implemented as a permissioned simulation ledger rather than a cryptocurrency-based consensus network. Each ledger entry stored the block index, timestamp, nonce, ciphertext, authentication tag, predicted label, anomaly score, key-version identifier, previous-block hash, and current-block hash. Ledger validation included authentication-tag verification, block-hash recomputation, and previous-hash linkage verification. Performance was evaluated using accuracy, detection rate, false-positive rate, precision, recall, F1-score, ROC–AUC, encryption and decryption time, hashing and verification time, block-creation time, transaction-verification time, throughput, latency, and resource utilization. All timing measurements were obtained under the same hardware and software configuration.

### 6.2. Transparent Comparative Evaluation

The classification component of the proposed framework, denoted as WSHB-DNN, was compared with LSTM and CNN–RNN baselines under a common binary-classification protocol. The cryptographic and permissioned-ledger components were evaluated separately because AES-256-GCM, hashing, and ledger logging protect confirmed attack-event records but do not modify the predicted class labels. Therefore, the comparison in this subsection focuses only on classifier performance.

For each dataset, all models used the same fixed sampled subset, the same 70:15:15 training, validation, and testing split, the same training-derived preprocessing parameters, and the same mutual-information-weighted 64-feature representation. HIKARI-2021 was evaluated as a network-flow intrusion-detection dataset, whereas ADFA-LD was evaluated as a host-based system-call intrusion-detection dataset. The final labels were predicted by the trained classifier; no signature-matching or rule-based classifier was used to determine the final class.

#### 6.2.1. Baseline Architecture and Tuning Protocol

Table 3 reports the complete baseline architectures and trainable parameter counts. WSHB-DNN used three dense hidden layers containing 128, 64, and 32 neurons. The LSTM baseline used one LSTM layer followed by a 32-neuron dense layer. The CNN–RNN baseline used a one-dimensional convolutional layer, max-pooling, a recurrent layer, and a 32-neuron dense layer.

All models were trained using Adam optimization, class-weighted binary cross-entropy, a batch size of 256, and early stopping. Each model was assigned the same search budget of three candidate configurations. The best configuration was selected using validation macro-F1. The decision threshold was also selected exclusively from the validation subset to maximize macro-F1. The final testing subset was used only once for the reported evaluation.

#### 6.2.2. Input and Sequence Construction

All models received the same fixed-length input representation. WSHB-DNN used the 64-dimensional weighted vector directly. For LSTM and CNN–RNN, the same vector was reshaped to (64,1). No cross-record temporal windows were constructed, and no model received additional sequential context from neighbouring samples. This design ensures that the comparison reflects model behavior under the same input information rather than differences in data representation.

For HIKARI-2021, numerical network-flow attributes were median-imputed, normalized using min–max scaling, and ranked using mutual information computed only from the training subset. For ADFA-LD, each system-call trace was first converted into a 3-g TF–IDF representation with a maximum vocabulary of 512 features. Training-only mutual-information ranking was then applied, and the top 64 weighted features were retained for classification, as shown in Table 4.

#### 6.2.3. Repeated-Run Classification Results

Final performance was measured over five independent initialization seeds: 42, 52, 62, 72, and 82. The sampled subset and data split were kept fixed across the repeated runs. Table 5 reports the mean and standard deviation for accuracy, attack-class F1-score, macro-F1, and ROC–AUC.

**Figure 3:**
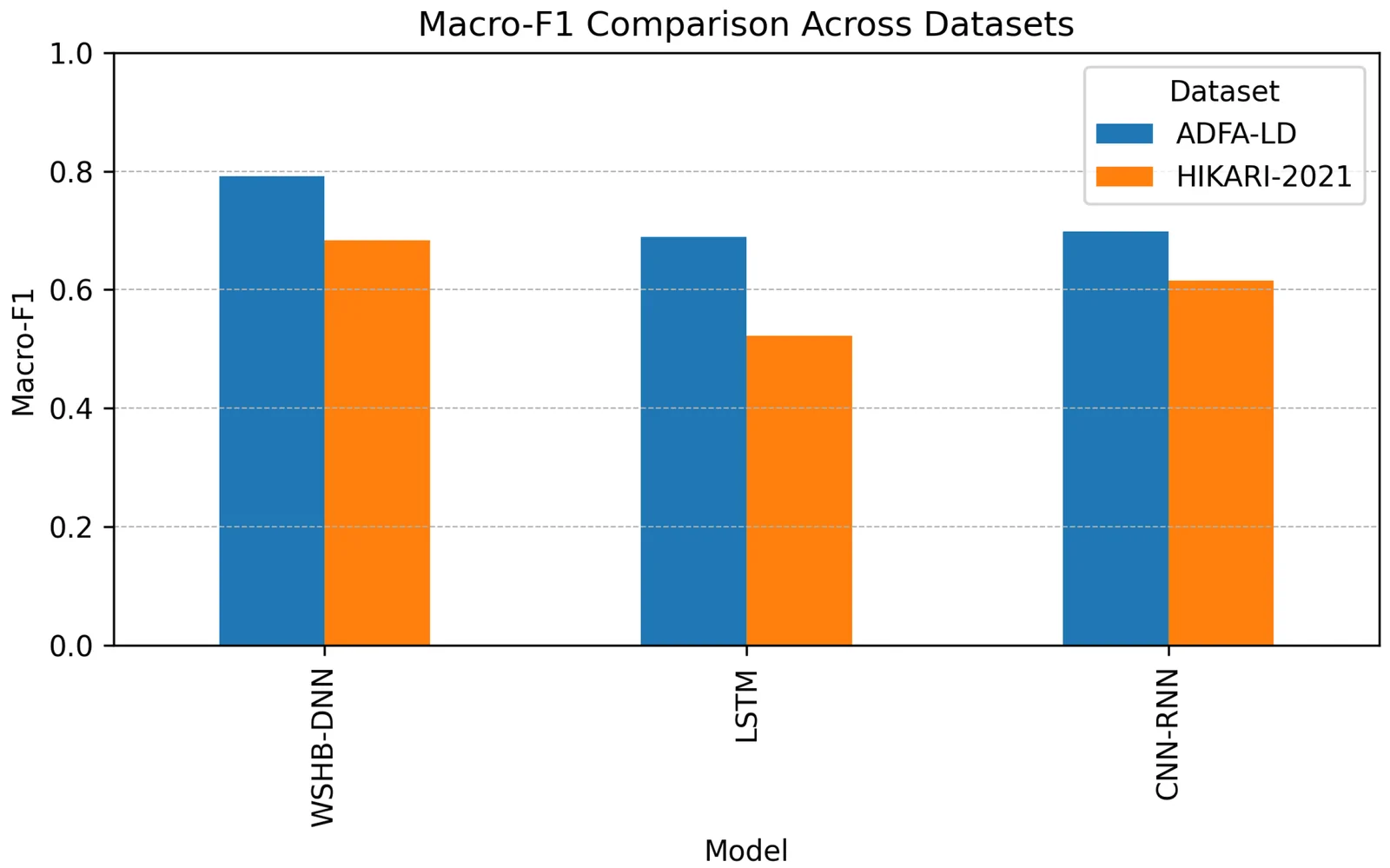
Macro-F1 comparison of WSHB-DNN, LSTM, and CNN–RNN on HIKARI-2021 and ADFA-LD.

**Figure 4:**
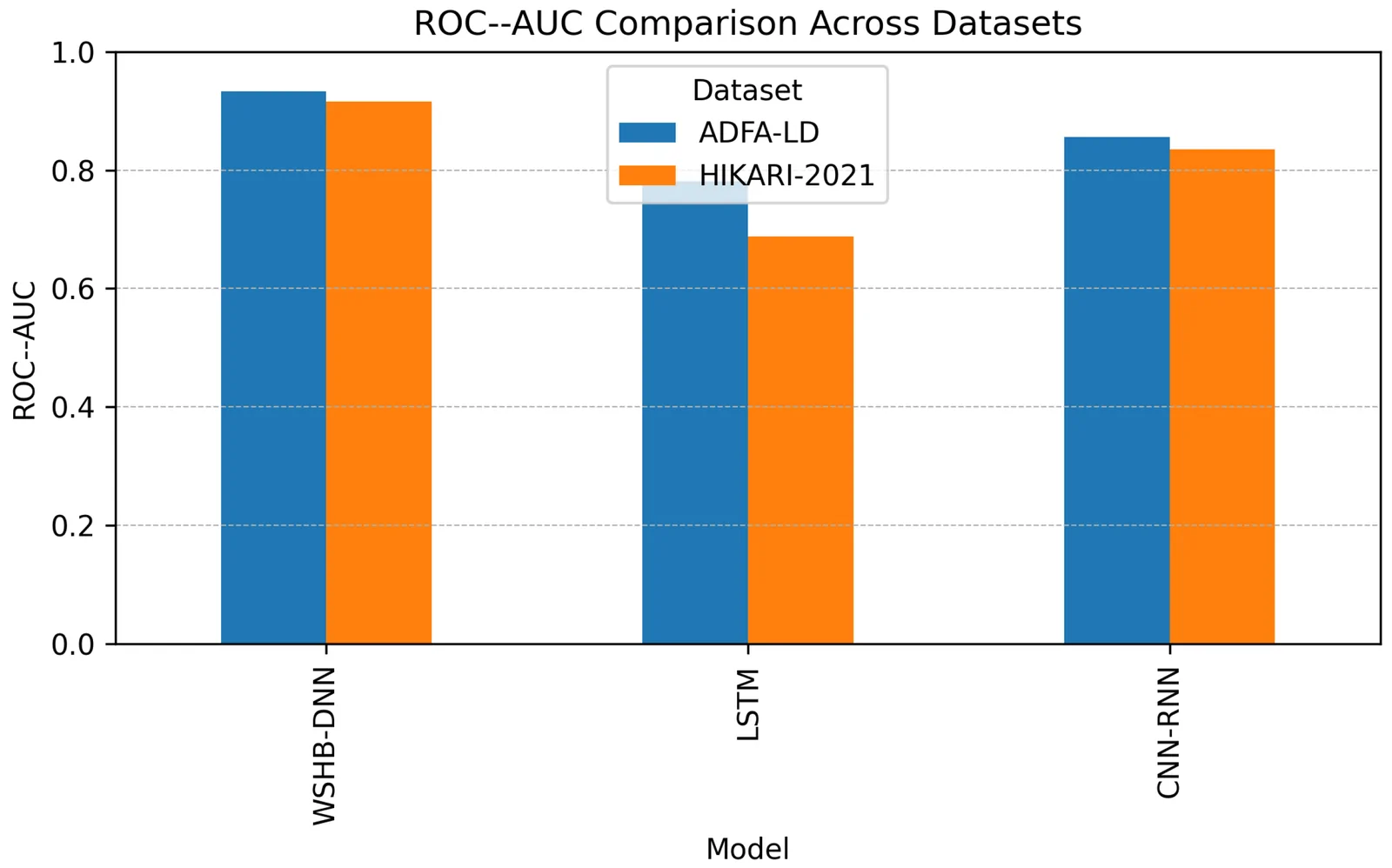
ROC–AUC comparison of WSHB-DNN, LSTM, and CNN–RNN on HIKARI-2021 and ADFA-LD.

Figure 3 compares macro-F1 across the two datasets, while Figure 4 compares ROC–AUC. The results show that WSHB-DNN achieved the highest mean macro-F1 and ROC–AUC on both datasets.

The repeated-run results demonstrate that WSHB-DNN provides the strongest overall classification performance under the selected experimental protocol. On HIKARI-2021, WSHB-DNN achieved the highest mean accuracy (0.8851±0.0103), attack-class F1-score (0.4314±0.0312), macro-F1 score (0.6837±0.0155), and ROC–AUC (0.9164±0.0082). On ADFA-LD, WSHB-DNN also achieved the highest mean accuracy (0.9091±0.0057), attack-class F1-score (0.6347±0.0292), macro-F1 score (0.7914±0.0159), and ROC–AUC (0.9328±0.0061), as shown in Table 6.

Although CNN–RNN obtained a slightly higher attack recall than WSHB-DNN on HIKARI-2021, WSHB-DNN achieved higher attack precision, attack F1-score, macro-F1, and ROC–AUC. Therefore, the observed advantage is reported as an overall classification improvement rather than superiority across every individual metric.

#### 6.2.4. Statistical Analysis

Paired statistical analysis was conducted using the macro-F1 scores from the five repeated runs. The paired *t*-test showed that WSHB-DNN achieved higher macro-F1 than LSTM and CNN–RNN on both datasets, with p<0.01 in all four comparisons. The Wilcoxon signed-rank test returned p=0.0625, which is close to but above the 0.05 significance threshold because only five paired runs were available. Therefore, the results support a consistent repeated-run improvement, while the non-parametric significance evidence should be interpreted cautiously, as shown in Table 7.

### 6.3. Cybersecurity Analysis with WSHB

This subsection analyzes the cybersecurity behavior of WSHB after the main comparative evaluation reported in Section 6.2. The comparative evaluation focuses on WSHB-DNN, LSTM, and CNN–RNN under a common binary protocol, whereas this subsection focuses on WSHB-specific cybersecurity visualization and post-detection protection. Therefore, the figures in this subsection report dataset-specific attack-family behavior and cryptographic processing rather than repeating the baseline comparison.

To avoid label inconsistency, HIKARI-2021 and ADFA-LD are visualized using their native class labels. HIKARI-2021 is represented using Background, Benign, Bruteforce, Bruteforce-XML, Probing, and XMRIGCC CryptoMiner classes. ADFA-LD is represented using Benign, Adduser, Hydra-FTP, Hydra-SSH, Java-Meterpreter, Meterpreter, and Webshell classes. Generic attack names such as DDoS, SQL injection, phishing, ransomware, cross-site scripting, and man-in-the-middle are not used for ADFA-LD because ADFA-LD is a host-based system-call dataset.

**Figure 5:**
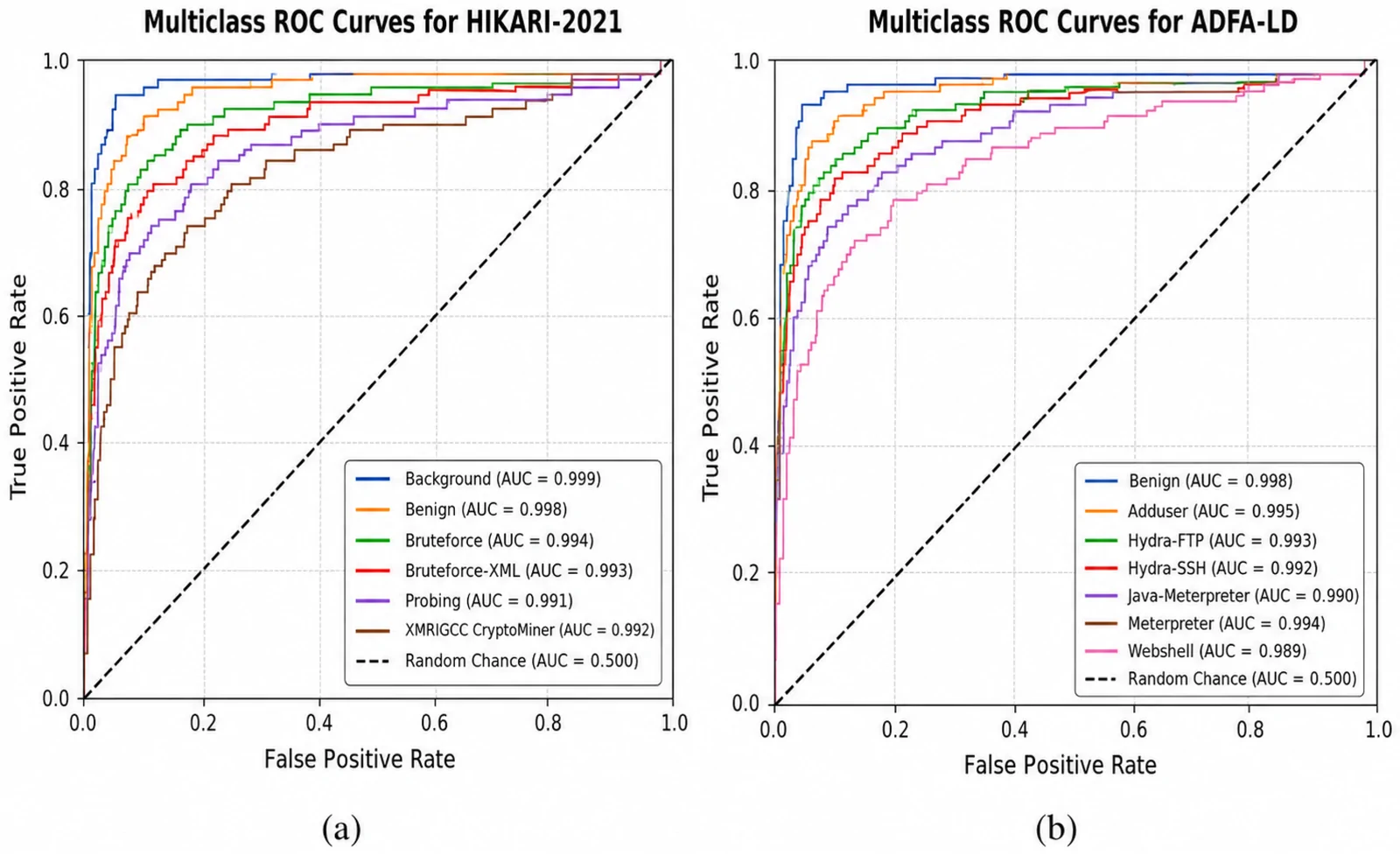
One-vs-rest multiclass ROC curves for (**a**) HIKARI-2021 and (**b**) ADFA-LD using dataset-specific class labels.

Figure 5 presents the one-vs-rest multiclass ROC curves for the two datasets. The curves show the class-wise discriminative behavior of WSHB under dataset-specific label settings. This visualization complements the binary repeated-run results reported in Section 6.2 by showing how the model separates individual attack families within each dataset.

**Figure 6:**
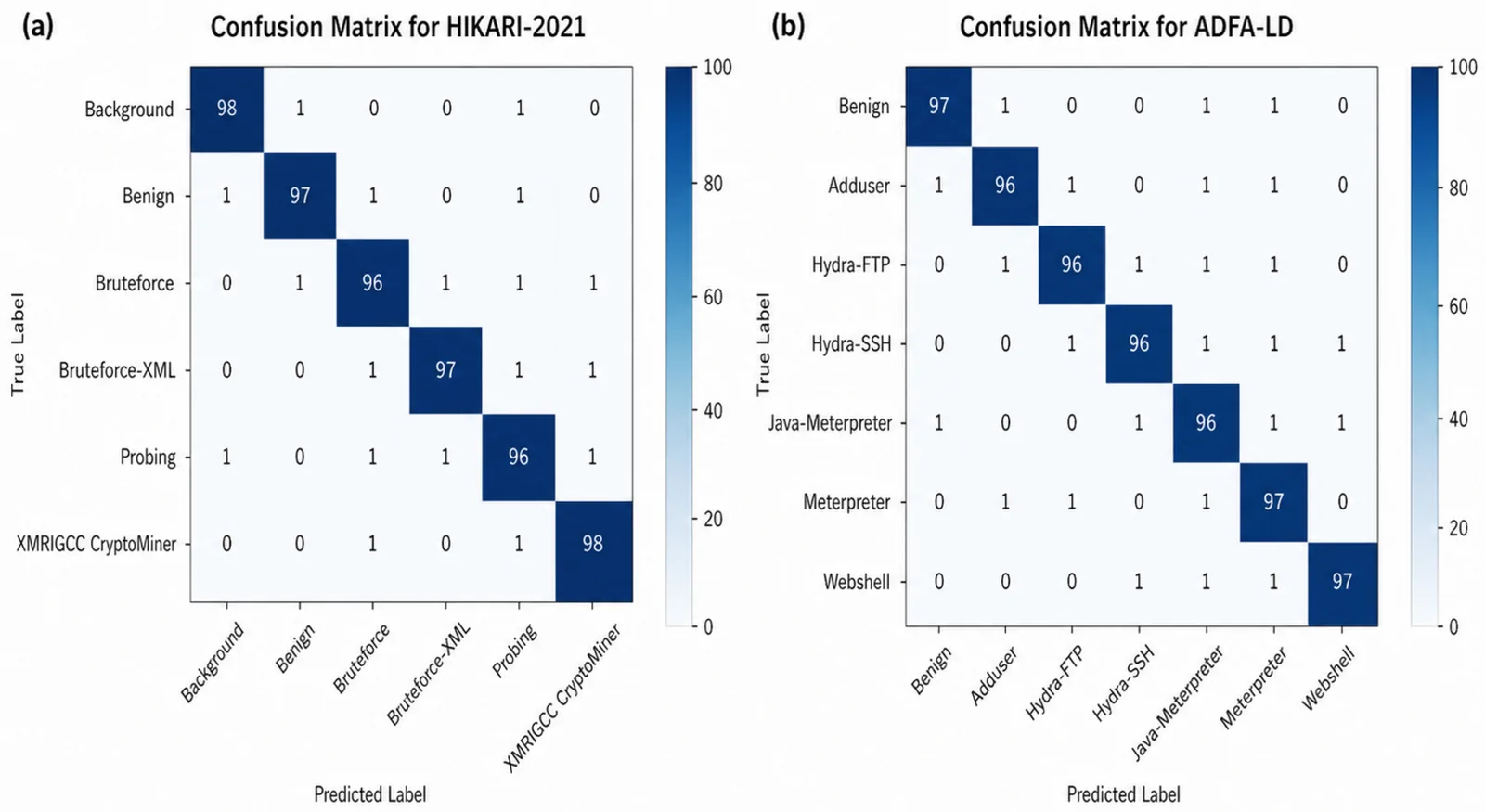
Multiclass confusion matrices for (**a**) HIKARI-2021 and (**b**) ADFA-LD using dataset-specific labels.

Figure 6 shows the corresponding multiclass confusion matrices. The diagonal values indicate correctly classified samples, while off-diagonal values indicate class-level confusion. The figure is used to identify which attack families are more likely to overlap in the learned feature space.

**Figure 7:**
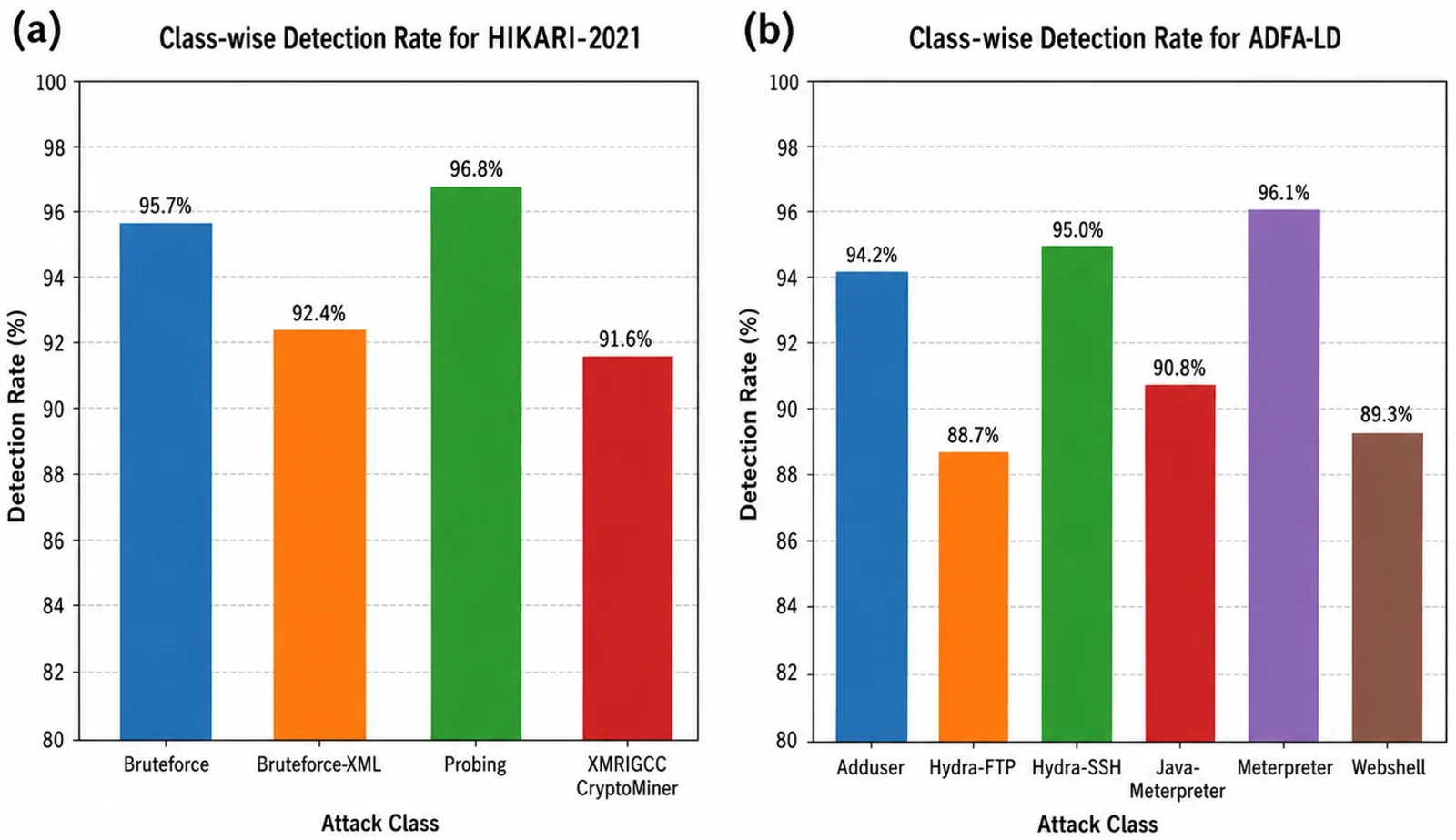
Class-wise detection rate for (**a**) HIKARI-2021 and (**b**) ADFA-LD.

Figure 7 reports the class-wise detection rate. For HIKARI-2021, the detection-rate analysis focuses on the attack classes Bruteforce, Bruteforce-XML, Probing, and XMRIGCC CryptoMiner. For ADFA-LD, the analysis focuses on Adduser, Hydra-FTP, Hydra-SSH, Java-Meterpreter, Meterpreter, and Webshell. This class-wise view helps identify attack families that are easier or more difficult for WSHB-DNN to detect.

**Figure 8:**
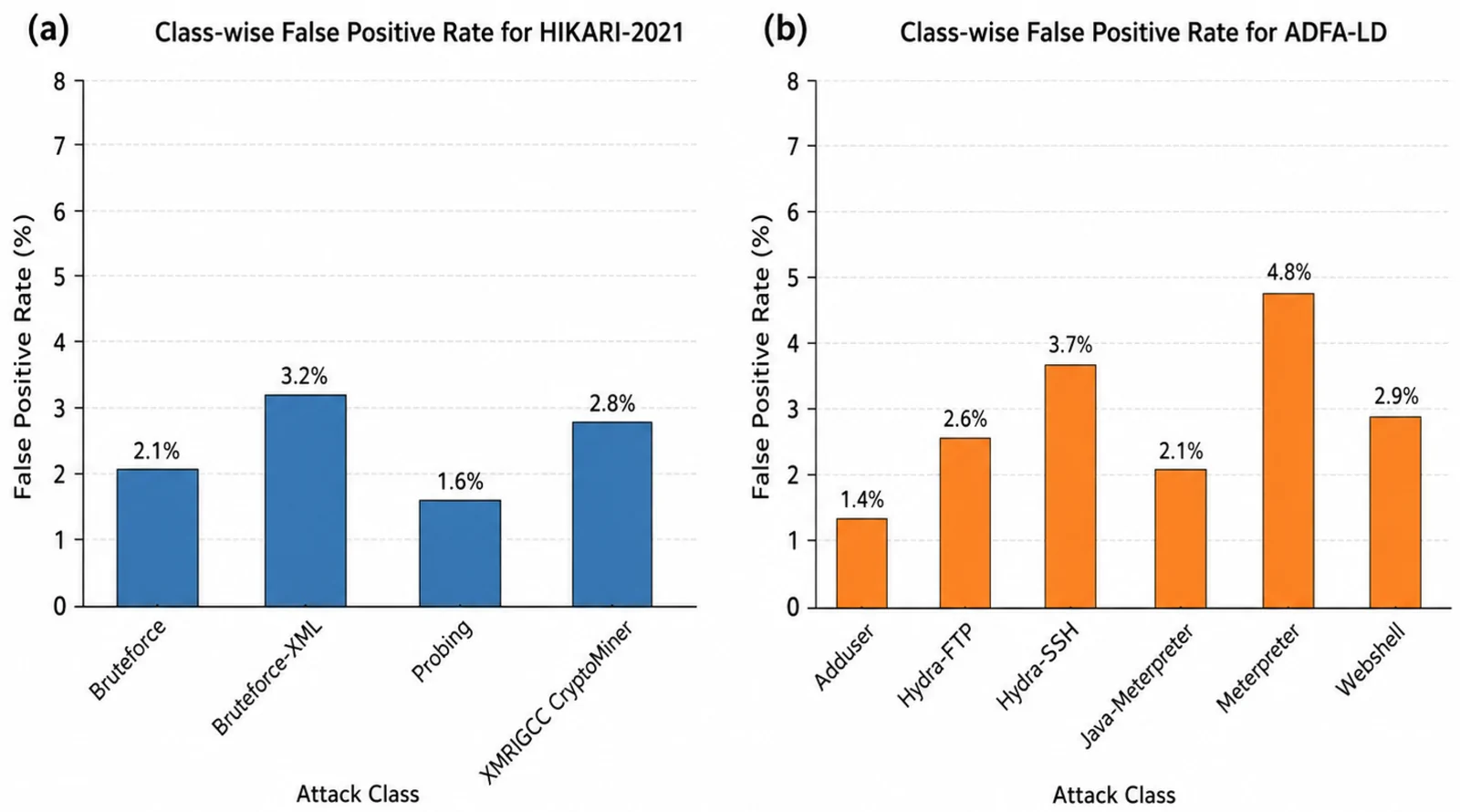
Class-wise false-positive rate for (**a**) HIKARI-2021 and (**b**) ADFA-LD.

Figure 8 reports the class-wise false-positive rate. Lower false-positive rates indicate fewer benign or non-target samples being incorrectly assigned to the corresponding attack class. This figure therefore complements the detection-rate analysis by showing the false-alarm behavior of WSHB at the class level.

**Figure 9:**
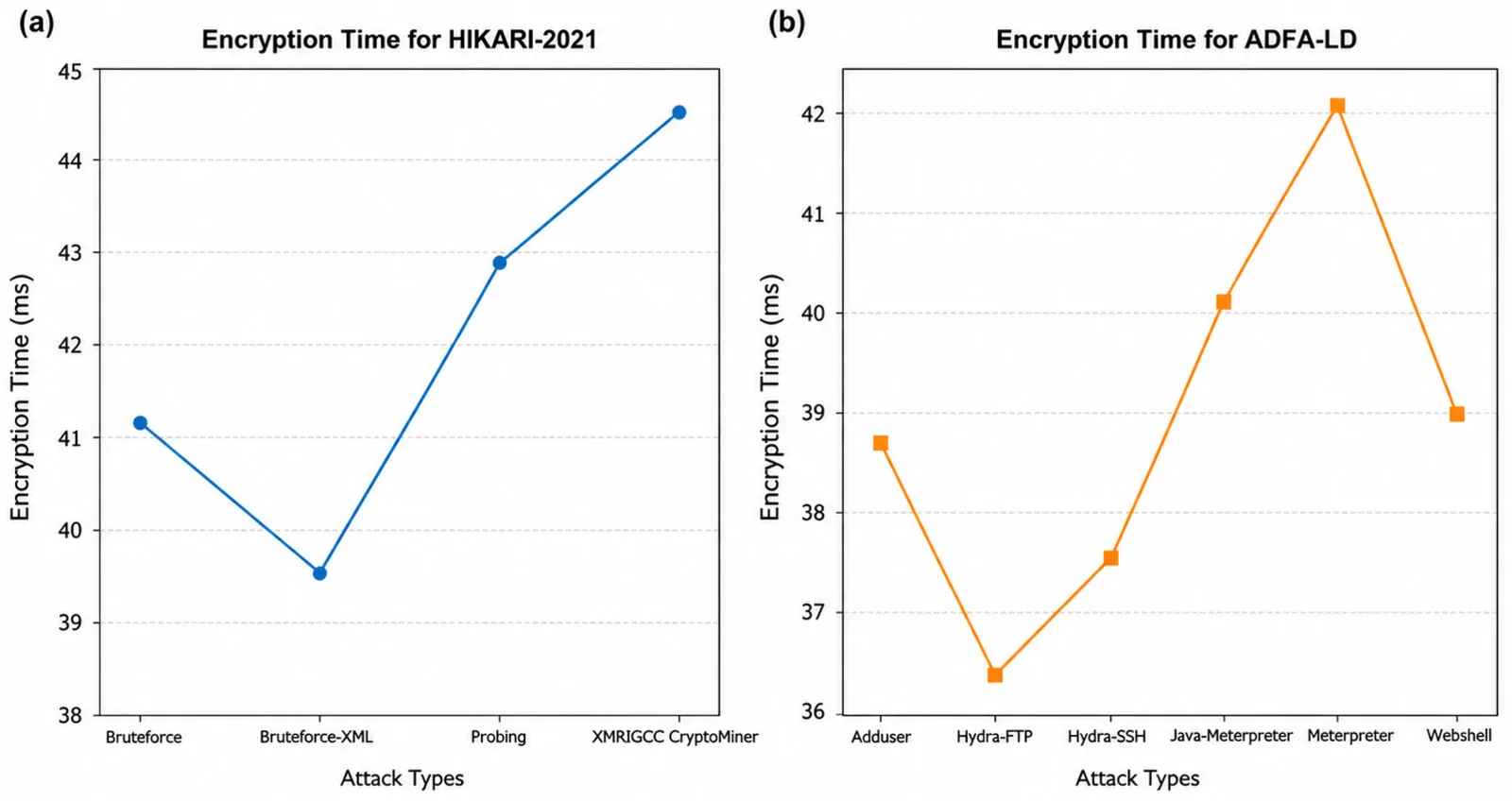
AES-256-GCM encryption time for representative confirmed attack-event categories in (**a**) HIKARI-2021 and (**b**) ADFA-LD.

After an attack event is confirmed by the DNN classifier, WSHB applies post-detection protection. Each confirmed attack-event record is encrypted using AES-256-GCM with a versioned 256-bit key, a fresh 96-bit nonce, and a 128-bit authentication tag. Figure 9 shows the encryption time across representative attack-event categories. The cryptographic processing is applied after classification and therefore does not directly change the predicted class label.

**Figure 10:**
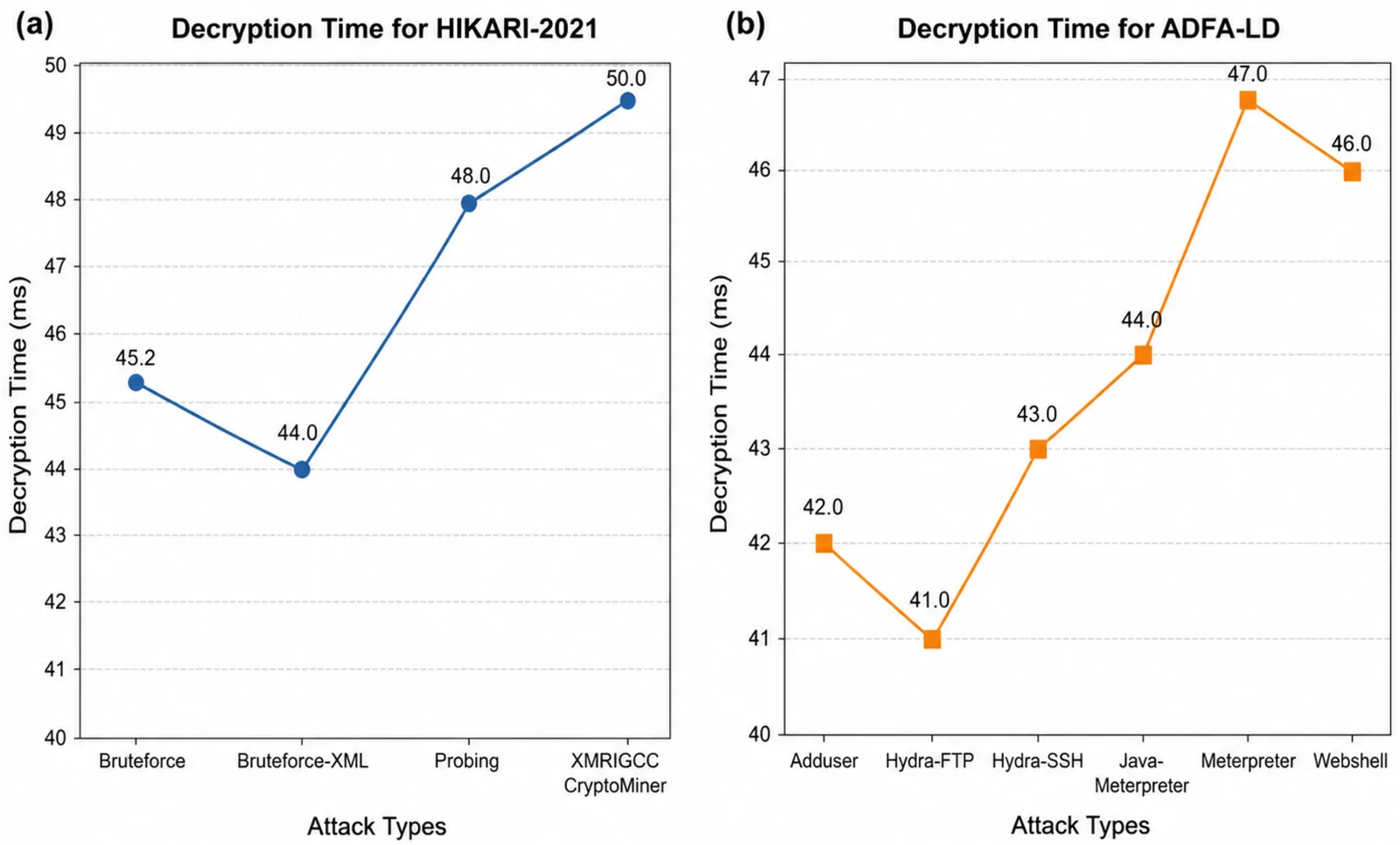
AES-256-GCM decryption time for representative confirmed attack-event categories in (**a**) HIKARI-2021 and (**b**) ADFA-LD.

Figure 10 reports the corresponding decryption time. During decryption, the AES-GCM authentication tag is verified before releasing the plaintext, which provides record-level authenticity and integrity. These results show the cryptographic overhead introduced by the post-detection protection stage.

After encryption, SHA-256 hashing is applied over the encrypted record and associated metadata. The resulting ledger entry stores the ciphertext, nonce, authentication tag, predicted label, anomaly score, key-version identifier, previous-block hash, and current-block hash. Ledger validation is performed through authentication-tag verification, block-hash recomputation, and previous-hash linkage verification. Thus, the classification component detects attacks, whereas the cryptographic and permissioned-ledger components provide confidentiality protection, integrity verification, authentication, and tamper-evident security-event logging.

## 7. Conclusions

This study proposed the Weighted Symmetric Hashed Blockchain (WSHB) framework for security monitoring in cloud-integrated sensor and IoT environments. The framework combines weighted feature analysis, AES-based symmetric encryption, cryptographic hashing, blockchain-based event logging, and deep learning-based attack classification. The proposed design aims to support confidentiality, integrity verification, tamper-evident logging, and attack classification within a unified workflow.

The framework was evaluated independently using HIKARI-2021 for network-based intrusion detection and ADFA-LD for host-based intrusion detection. Under the documented repeated-run protocol, WSHB-DNN achieved macro-F1 scores of 0.6837 on HIKARI-2021 and 0.7914 on ADFA-LD, showing the strongest overall classification performance among the evaluated models. The cryptographic and permissioned-ledger components were evaluated as post-detection protection mechanisms that provide confidentiality, record-level authentication, integrity verification, and tamper-evident logging for confirmed attack-event records.

This work does not claim full real-world scalability or complete real-time protection, as large-scale deployment and live network validation were outside the scope of the current evaluation. Future work will focus on validating WSHB in real cloud-IoT testbeds, evaluating the framework against additional benchmark datasets, optimizing blockchain overhead, and conducting broader statistical and baseline comparisons under diverse attack scenarios.

## Figures and Tables

**Table 1:** Dataset and subset configuration.

Configuration	HIKARI-2021	ADFA-LD
Detection setting	Network-based IDS	Host-based IDS
Data representation	Network-flow records	System-call traces
Selected subset	10,000 records	5000 traces
Sampling method	Stratified random sampling	Stratified random sampling
Sampling seed	42	42
Binary label mapping	0: benign; 1: attack	Normal traces: benign; attack-directory traces: attack
Feature processing	Categorical encoding and min–max normalization	Fixed-length numerical representation of system-call traces
Data split	70% training, 15% validation, and 15% testing	70% training, 15% validation, and 15% testing

**Table 2:** Dataset-specific experimental and cryptographic configuration of the WSHB framework.

Parameter	HIKARI-2021	ADFA-LD
Intrusion-detection setting	Network-based intrusion detection	Host-based intrusion detection
Dataset source	ALLFLOWMETER_HIKARI2021.csv	Official ADFA-LD archive
Data representation	Labelled network-flow records	Ordered Linux system-call traces
Selected subset	10,000 network-flow records	5000 system-call traces
Sampling method	Stratified random sampling	Stratified random sampling
Sampling seed	42	42
Binary label mapping	Label 0: benign; label 1: attack	Normal traces: benign; attack-directory traces: attack
Feature processing	Categorical encoding and min–max normalization	Fixed-length numerical representation of system-call traces
Data split	70% training, 15% validation, and 15% testing	70% training, 15% validation, and 15% testing
Authenticated encryption	AES-256-GCM	AES-256-GCM
Encryption-key length	256 bits	256 bits
Nonce/IV	Fresh and unique 96-bit nonce per encrypted record	Fresh and unique 96-bit nonce per encrypted record
Ledger hashing	SHA-256	SHA-256
Authentication tag	128-bit AES-GCM authentication tag	128-bit AES-GCM authentication tag
Key management	Access-controlled and versioned keys	Access-controlled and versioned keys
Key rotation	Fresh key version for each independent run	Fresh key version for each independent run
Key revocation	Compromised key version disabled and replaced	Compromised key version disabled and replaced
Blockchain type	Permissioned simulation ledger	Permissioned simulation ledger
Ledger validation	Authentication-tag verification, block-hash recomputation, and previous-hash linkage verification	Authentication-tag verification, block-hash recomputation, and previous-hash linkage verification

**Table 3:** Architectures and trainable parameter counts used in the comparative evaluation.

Dataset	Model	Architecture	Parameters
HIKARI-2021	WSHB-DNN	Input(64)–Dense(128)–Dense(64)–Dense(32)–Sigmoid; dropout = 0.3	18,689
HIKARI-2021	LSTM	Input(64, 1)–LSTM(32)–Dense(32)–Sigmoid; dropout = 0.2	5569
HIKARI-2021	CNN–RNN	Input(64, 1)–Conv1D(64, *k* = 3)–MaxPool–RNN(64)–Dense(32)–Sigmoid; dropout = 0.3	10,689
ADFA-LD	WSHB-DNN	Input(64)–Dense(128)–Dense(64)–Dense(32)–Sigmoid; dropout = 0.2	18,689
ADFA-LD	LSTM	Input(64,1)–LSTM(32)–Dense(32)–Sigmoid; dropout = 0.2	5569
ADFA-LD	CNN–RNN	Input(64, 1)–Conv1D(32, *k* = 3)–MaxPool–RNN(32)–Dense(32)–Sigmoid; dropout = 0.2	3329

**Table 4:** Hyperparameter search ranges and selection protocol.

Model	Candidate Values and Selection Rule
WSHB-DNN	Dropout ∈{0.2,0.3} and learning rate $\in \{10^{-3},5\times 10^{-4}\}$. Three candidate configurations were tested, and the configuration with the highest validation macro-F1 was selected.
LSTM	LSTM units ∈{32,64}, dropout ∈{0.2,0.3}, and learning rate $\in \{10^{-3},5\times 10^{-4}\}$. Three candidate configurations were tested, and the configuration with the highest validation macro-F1 was selected.
CNN–RNN	Convolution filters ∈{32,64}, kernel size ∈{3,5}, RNN units ∈{32,64}, dropout ∈{0.2,0.3}, and learning rate $\in \{10^{-3},5\times 10^{-4}\}$. Three candidate configurations were tested, and the configuration with the highest validation macro-F1 was selected.

**Table 5:** Binary classification results over five independent initialization seeds, reported as mean ± standard deviation.

Dataset	Model	Accuracy	Attack F1	Macro-F1	ROC–AUC
HIKARI-2021	WSHB-DNN	0.8851 ± 0.0103	0.4314 ± 0.0312	0.6837 ± 0.0155	0.9164 ± 0.0082
HIKARI-2021	LSTM	0.7561 ± 0.0975	0.1917 ± 0.0953	0.5218 ± 0.0448	0.6883 ± 0.0613
HIKARI-2021	CNN–RNN	0.8237 ± 0.0213	0.3320 ± 0.0577	0.6150 ± 0.0263	0.8358 ± 0.0454
ADFA-LD	WSHB-DNN	0.9091 ± 0.0057	0.6347 ± 0.0292	0.7914 ± 0.0159	0.9328 ± 0.0061
ADFA-LD	LSTM	0.8683 ± 0.0146	0.4541 ± 0.0249	0.6896 ± 0.0158	0.7814 ± 0.0209
ADFA-LD	CNN–RNN	0.8528 ± 0.0446	0.4823 ± 0.0449	0.6979 ± 0.0360	0.8564 ± 0.0289

**Table 6:** Per-class performance averaged over five independent runs.

Dataset	Model	Class	Precision	Recall	F1-Score	Support
HIKARI-2021	WSHB-DNN	Benign	0.9678 ± 0.0075	0.9065 ± 0.0168	0.9360 ± 0.0064	1392
HIKARI-2021	WSHB-DNN	Attack	0.3366 ± 0.0208	0.6093 ± 0.0992	0.4314 ± 0.0312	108
HIKARI-2021	LSTM	Benign	0.9496 ± 0.0141	0.7796 ± 0.1197	0.8518 ± 0.0681	1392
HIKARI-2021	LSTM	Attack	0.1269 ± 0.0516	0.4537 ± 0.2426	0.1917 ± 0.0953	108
HIKARI-2021	CNN–RNN	Benign	0.9674 ± 0.0186	0.8391 ± 0.0365	0.8980 ± 0.0148	1392
HIKARI-2021	CNN–RNN	Attack	0.2290 ± 0.0309	0.6259 ± 0.2187	0.3320 ± 0.0577	108
ADFA-LD	WSHB-DNN	Benign	0.9474 ± 0.0067	0.9488 ± 0.0067	0.9481 ± 0.0032	656
ADFA-LD	WSHB-DNN	Attack	0.6393 ± 0.0249	0.6319 ± 0.0508	0.6347 ± 0.0292	94
ADFA-LD	LSTM	Benign	0.9201 ± 0.0037	0.9302 ± 0.0199	0.9250 ± 0.0091	656
ADFA-LD	LSTM	Attack	0.4811 ± 0.0637	0.4362 ± 0.0361	0.4541 ± 0.0249	94
ADFA-LD	CNN–RNN	Benign	0.9312 ± 0.0076	0.8985 ± 0.0611	0.9135 ± 0.0302	656
ADFA-LD	CNN–RNN	Attack	0.4590 ± 0.0983	0.5340 ± 0.0794	0.4823 ± 0.0449	94

**Table 7:** Paired significance tests on macro-F1 across the five repeated runs.

Dataset	Comparison	Mean Difference	Paired *t*-Test *p*	Wilcoxon *p*
HIKARI-2021	WSHB-DNN vs. LSTM	0.1619	0.0008163	0.0625
HIKARI-2021	WSHB-DNN vs. CNN–RNN	0.0687	0.001261	0.0625
ADFA-LD	WSHB-DNN vs. LSTM	0.1018	0.0009668	0.0625
ADFA-LD	WSHB-DNN vs. CNN–RNN	0.0935	0.008857	0.0625

## Data Availability

The datasets analyzed in this study are publicly available on Kaggle: the HIKARI-2021 PCAP Dataset [26], available at https://www.kaggle.com/datasets/ingenio/hikari2021-pcap (accessed on 8 June 2026); and the ADFA-LD Dataset [27], available at https://www.kaggle.com/datasets/yianqaq/adfa-ld (accessed on 8 June 2026).
